# A narrative review of precision and ethical considerations in cardiovascular health: CRISPR-Cas9, telemedicine, and lifestyle interventions

**DOI:** 10.3389/fpubh.2025.1737251

**Published:** 2026-01-21

**Authors:** Zhoumin Lu, Syeda Taqveem Hassan Bukhari, Muhammad Azeem, Nusrat Tariq, Muhammad Abu Bakr Shabbir

**Affiliations:** 1 Department of Cardiovascular Medicine, Dazhou Dachuan District People’s Hospital China (Dazhou Third People’s Hospital), Dazhou, China; 2 Institute of Microbiology, University of Veterinary and Animal Sciences, Lahore, Pakistan; 3 Department of Allied Health Sciences, The Superior University, Lahore, Pakistan; 4 M. Islam Medical and Dental College, Gujranwala, Pakistan

**Keywords:** behavioral change, CRISPR-Cas9, CVDs, precision medicine, public health, risk factor

## Abstract

**Background:**

Cardiovascular diseases (CVDs) remain the leading cause of global morbidity and mortality, influenced by lifestyle, socioeconomic status, and genetic factors. Emerging innovations, including wearable health technologies, telemedicine, and CRISPR-Cas9 gene editing, provide new possibilities for rapid prevention and personalized management.

**Methods:**

This narrative review collected evidence from Scopus, PubMed, and Google Scholar, using keywords such as cardiovascular (CV) prevention, lifestyle determinants, digital health, telemedicine, CRISPR-Cas9, and public health ethics. Eligible peer-reviewed studies, clinical guidelines, and policy documents were included to assess behavioral, technological, and genomic strategies for CVD care.

**Results:**

Modifications in lifestyle, such as quitting smoking, regular physical activity, following a heart-healthy diet plan, and getting adequate sleep, can significantly reduce the risk of CVD. Additionally, telemedicine and wearable devices facilitate early detection, better self-management, and treatment adherence, especially in underserved communities. CRISPR-Cas9 holds a significant potential for correcting genetic variants related to lipid disorders and inherited cardiomyopathies, but its clinical translation remains in early stages. However, existing evidence is limited by heterogeneity in study design, brief follow-up, particularly for digital health and CRISPR applications. Additional challenges, such as health inequities, digital access, data privacy, and ethical oversight, further influence their real-world implementation.

**Conclusion:**

Effective integration of behavioral, digital and genomic innovations requires policy frameworks that ensure equity, ethical governance, and long-term sustainability. Combining precision medicine with efforts to address social determinants of health will be crucial in reducing the global burden of CVD and shaping the future of CV care.

## Introduction

The cardiovascular system is the first organ that develops during embryogenesis, ensuring not only the transport of nutrients and oxygen but also the removal of metabolic waste from the very beginning of life (1). From the first to the last heartbeat, the said system functioning is vital as any abnormality (both functional and structural) could have a life-threatening situation.

The prevalence of CVD has doubled worldwide in the last 20 years, reaching over 500 million cases in 2019 alone, highlighting the growing public health concern. In addition, the World Health Organization (WHO) reported that CVD alone is responsible for one-third of all deaths globally (1, 2). According to the WHO, CVD accounts for about 17.9 million deaths annually, making it the leading cause of mortality worldwide (3). Likewise, the Centers for Disease Control and Prevention (CDC) in 2021 reported that CVD is the leading cause of death in the United States (US) (4). The American Heart Association (AHA) reports that CVDs affect more than 26 million people in the US, and the associated annual cost is expected to escalate from USD 216 billion in 2016–2017 to USD 749 billion by the year 2035 (5, 6). These statistics highlight the substantial economic and social implications of CVD on global and national healthcare systems.

Even with significant advancements in CV medicine, CVD is still a multifactorial disorder influenced by behavioral factors, such as smoking, drinking, physical inactivity, inadequate sleep, and dietary behavior. Social determinants include psychological stress, ethnicity, food scarcity, and the unavailability of quality healthcare facilities. Biological determinants include family history, hypertension, metabolic syndrome, and chronic kidney disease are among the modifiable and non-modifiable determinants identified by the AHA (7). Collectively, these risk factors contribute to more than 90% of CVD cases worldwide, highlighting the significance of integrated prevention strategies (8).

A major factor in the progression and prevention of CVD is dietary behavior. According to early epidemiological studies, individuals who consume diets high in grains, fruits, legumes, vegetables, and fish have lower rates of myocardial infarction (MI) compared to those who consume diets high in animal fat (9). Additionally, according to the Prospective Urban Rural Epidemiology (PURE) study, conducted in 18 different countries, people with high intake of fruits, vegetables, and legumes exhibited a 19% reduction in the all-cause mortality rate. However, people with high intake of processed foods, trans fats, and sugar-sweetened beverages are more prone to CVD. Therefore, it is necessary to eat a plant-based, Mediterranean, or Dietary Approaches to Stop Hypertension (DASH) diet, as these diets are cardio-protective (10). Evidence from multinational cohort data further confirms that Mediterranean and DASH diets consistently reduce CVD risk (11). Emerging research also suggested that diet-induced modulation of the gut microbiota may affect cardiometabolic outcomes, opening up new possibilities for personalized nutrition-based prevention.

In addition to biological and lifestyle determinants, CV outcomes are significantly influenced by social and structural factors collectively known as social determinants of health (SDoH). Furthermore, urbanization, healthcare access, lower income, and educational attainment all have a significant impact on the incidence and prognosis of CVD. Individuals from lower socioeconomic backgrounds frequently experience chronic psychosocial stress, limited availability of healthy food, and unavailability of good healthcare facilities, leading to the increased risk of CVD (3). Similarly, people with a low literacy rate ultimately have little knowledge about health and preventive measures (12). Additionally, urbanization is a significant risk factor for air pollution and thus plays a vital role in the high prevalence of CVD (13). Inequities in access to primary healthcare are also a contributing factor to disease management and long-term outcomes (14). Addressing these social and structural inequities aligns with the WHO’s global health equity framework, which emphasizes emphasizing fair and universal access to preventive CV services (15).

Taken together, these behavioral, dietary and socioeconomic determinants highlight the broad population-level contributors to CV risk. Although lifestyle modification remains foundational, advances in digital health like telemedicine, wearable sensors and mobile health applications now play a significant role in early detection, treatment adherence, and remote management of CVD risk factors (16–18). These technologies can improve access and enhance preventive services to geographically remote communities. Telemedicine systems enhance access to care for patients by reducing blood pressure and cholesterol levels in underserved and rural areas through regular virtual monitoring and counseling (19). Digital health technologies, such as wearable sensors and mobile apps, enable the continuous monitoring of vital signs and behavioral habits, facilitating timely clinical interventions (20). In low and middle-income countries (LMI), there have been quantifiable improvements in lifestyle change, drug adherence, and blood pressure control through community CV prevention programs (21). These innovations enable cost-efficient, scalable, and patient-centered care models that address healthcare delivery gaps across various socioeconomic environments (22). However, access to such technologies remains uneven as digital literacy, data privacy, internet limitations, device costs and infrastructural inequalities need to be addressed so that such technological advancements promote health equity rather than exacerbating existing disparities (23).

Extending these behavioral and digital approaches, community-based CV prevention programs in LMI showed promising improvements in lifestyle behaviors, medication adherence and blood pressure control (21). Although behavioral and digital approaches improve the CV prevention at the individual level, emerging genomic innovations like Clustered Regularly Interspaced Short Palindromic Repeats (CRISPR)-Cas9 gene editing system provide additional opportunities to precisely target the inherited contributors of CVD (24, 25).

This narrative review aims to synthesize the current evidence on lifestyle determinants, innovations in digital health, and genomic advancements like CRISPR-Cas9 technologies related to CV health, emphasizing their public health implications, ethical considerations and potential to reduce the global burden of CVD. Specifically, we explore key behavioral and dietary risk factors, evaluate evidence-based lifestyle and nutrition interventions, and highlight the socioeconomic determinants like food insecurity and health inequities that impede heart health practices. The review also discusses the emerging genomic tool’s role in correcting the disease-related variants within the precision medicine (evidence based clinical approach that integrates individual genetic, molecular information to guide personalized disease prevention, diagnosis and treatment) framework. Lastly, ethical challenges associated with behavioral, digital and genomic advancements were also discussed in this review.

## Methods

This narrative review collected evidence on behavioral determinants and advancements in digital health and genomic approaches related to CVD prevention and management. A structured and transparent approach was adopted in the selection and identification of literature.

### Literature search strategy

A comprehensive search was performed across Google Scholar, PubMed and Scopus. These three databases were selected to cover a wide range of clinical, behavioral, public health and genomic studies related to CV health.

The keywords used for search in these databases were CVD prevention, lifestyle modification, CVD risk factors, socioeconomic status, dietary intervention, digital health, telemedicine, wearable sensors, genomic advancements, CRISPR-Cas9, gene editing and lastly public health ethics. Boolean operators, such as AND/OR, were used to refine the search to ensure the detailed coverage of thematic areas.

### Inclusion criteria

The inclusion criteria for studies were (i) peer-reviewed, (ii) focused on behavioral, (iii) dietary, (iv) telemedicine, (v) digital health, (vi) genomic approaches to CVD, (vii) reported clinical, public health and translational outcomes, (viii) original research articles, meta-analysis, systematic review, guideline documents and authoritative policy reports, and lastly published in English.

### Exclusion criteria

The exclusion criteria for studies include, (i) reports exclusively on animal models without any translational relevance, (ii) did not address CV outcomes, (iii) conference abstracts, letters, editorials, non-peer-reviewed commentary, and (iv) were not published in English.

### Study screening and selection

Initially, a broad set of publications was retrieved across three databases. Titles and abstracts were screened to determine the relevance to CVD prevention and thematic domains of lifestyle, telemedicine, digital health and genomics. The selected articles were further evaluated according to the predefined guidelines of inclusion and exclusion criteria. Studies that seems topically relevant and methodologically sound were included in the final narrative review synthesis.

### Risk factors contributing to the development of cardiovascular disease: risk assessment

The assessment of CV risk is fundamentally based on an individual’s complete exposure to both modifiable and non-modifiable factors. Standardized risk assessment instruments are fundamental to preventive cardiology and are supported by both American and European recommendations. The European Society of Cardiology (ESC) endorses the Systematic Coronary Risk Evaluation (SCORE) tool, whereas the AHA and American College of Cardiology (ACC) advocate for the Atherosclerotic Cardiovascular Disease (ASCVD) risk calculator. These tools, freely available online, are validated for distinct populations-SCORE for European cohorts and ASCVD for US cohorts (26).

The predictive validity of CV risk models significantly differs among ethnic populations and in low-and middle–income countries (LMICs), where socioeconomic factors, dietary diversity, and healthcare access have a significant impact on CV outcomes (27). Conventional models like Framingham Risk Score and SCORE frequently miscalculate risk in non-Western populations due to regional variations in genetics, environment, and lifestyle (28). Incorporating social and behavioral determinants such as educational attainment, employment status, health literacy, and psychosocial stress into the current algorithms could significantly enhance predictive accuracy and increase policy relevance (29). These multifaceted approaches correspond with global health priorities focused on advancing equitable and contextually relevant CV prevention and interventions (30).

Behavioral modification remains a key factor in the prevention of CVD, as previously described in the ACC/AHA prevention guidelines (8). However, sustained behavioral change is largely influenced by environmental, socioeconomic and cultural factors rather than by individual willpower alone, as illustrated in a previous study (31). Although quitting smoking, following healthy diet plan, regular exercise, adequate sleep, and stress reduction can substantially reduce CVD risk (32). These behaviors are greatly influenced socioeconomic factors, such as income, neighborhood safety, surplus food and health literacy (33, 34). Evidence further suggested that behavioral interventions work best when embedded within supportive systems like community initiatives, nutrition policies, primary care counseling, and build environments that facilitate physical activity, rather than delivered in isolation (35, 36). Hence, integrating these behavioral interventions within the public health framework is necessary to achieve log-term and equitable reduction in CVD risk (37).

Behavioral Determinants

### Smoking and electronic cigarettes

Cigarette smoking is a leading preventable factor contributing to CV morbidity and mortality globally (8, 26). Approximately 1 billion individuals worldwide smoke, with around 12% of all CVD-related fatalities directly linked to tobacco exposure. There is no established safe threshold for smoking; both active and passive exposure markedly increase the risk of CVD. Electronic cigarettes (ECs) have become increasingly popular as an alternative to traditional smoking; however, the long-term CV safety of these devices is still uncertain. Recent evidence suggests that the use of ECs is associated with endothelial dysfunction, platelet activation, and systemic inflammation, all of which are factors that increase CV risk (38).

Effective tobacco control policies, such as taxation, smoke-free public spaces, behavioral counseling, and pharmacotherapy, significantly reduce the global burden of CVD (39). Research consistently shows that increased excise taxes and public smoking bans result in notable reductions in tobacco consumption and associated CVD morbidity and mortality (40). Behavioral counseling and pharmacological cessation aids, including nicotine replacement therapy and varenicline, significantly improve quit rates, especially when incorporated into primary health care systems (40). The increasing use of ECs among young adults and adolescents poses serious challenges to regulatory authorities in controlling the rapid adoption of ECs. Strengthening the ECs regulations, covering product safety, taxation, and marketing restrictions, has become a key component in protecting public health (41). These integrated strategies support the WHO Framework Convention on Tobacco Control, and promote a tobacco-free generation and thereby reduce the global incidence of CVD.

### Body mass and overweight conditions

Individuals with obesity [body mass index (BMI) ≥ 30 Kg/m^2^] and overweight (BMI ≥ 25 Kg/m^2^) are established known risk factors for type 2 diabetes, hypertension and CVD (42, 43). Maintaining body weight within a healthy range (BMI 20–24.9 Kg/m^2^) is associated with lower blood pressure, maintaining glycemic control, and a reduced risk of CVD. According to the latest study findings, relationship between obesity and CVD is multifaceted, involving factors such as BMI, systemic inflammation, visceral obesity, and metabolic dysregulation. It has been reported that visceral adiposity exhibits a stronger association with atherosclerosis, endothelial dysfunction, and insulin resistance than overall obesity, highlighting the importance of developing improved CV risk assessment models (44). Adipokines, such as tumor necrosis factor-*α* and interleukin-6, play a significant role in the development of cardiometabolic complications (45). Obesity related CV risks are further intensified by socioeconomic factors, including restricted availability of healthy food, urbanization, and polluted environments for physical activity, which exacerbate CVD risks, especially in LMI. A recent study reported that community-based programs, promoting balanced diets and regular physical activity, can substantially decrease morbidity and mortality associated with obesity-induced CVD (46). Therefore, implementing strategies that are both economically and culturally feasible can play an effective role in addressing the global cardiometabolic epidemic.

### Alcohol consumption

Alcohol is socially accepted in several cultures; however, its CV effects are contingent upon dosage. Moderate consumption may provide minimal advantages in certain groups; however, excessive alcohol intake (>100 g/week) elevates the risk of atrial fibrillation, hypertensive heart disease and stroke (47, 48).

Public health policies. Including alcohol taxation, community level awareness campaigns (organized interventions implemented within a specific population to promote health, and address social and behavioral needs) and marketing restrictions, are crucial in reducing excessive alcohol consumption and its related CV impacts (49). Epidemiological studies indicate a definitive dose–response relationship between alcohol consumption and the incidence of hypertension, cardiomyopathy, and atrial fibrillation, with moderate intake also associated with increased CVD risk (50). Policy frameworks that integrate taxation, limited sale hours, and advertising restrictions have demonstrated effectiveness in decreasing per capita alcohol consumption and associated hospital admissions (51). Implementation gaps persist in LMI, largely due to inadequate regulatory enforcement and industry interference (41). The WHO’s sustainable development goal (SDG 3.5) emphasizes the need to enhance global initiatives aimed at preventing excessive alcohol consumption, acknowledging its significant association with non-communicable diseases, such as CVD (52). To achieve these goals, it is essential to implement coordinated, evidence-based interventions that integrate regulatory, community and health care strategies.

### Hereditary background and genetic susceptibility

An individual having a family history of CVD can significantly increase individual risk, particularly when early-onset disease is present in first-degree relatives (53). Genetic predispositions also play a vital role in influencing lipid metabolism, regulating blood pressure and contributing to atherosclerosis.

Recent genomic studies have identified multiple loci linked to increased susceptibility to CVD, offering essential insights into the biological mechanisms involved in hypertension, atherosclerosis and dyslipidemia (54). Genome-wide association studies (GWAS) have identified that common variants in genes like APOE, PCSK9, and SORT1 significantly affect lipid metabolism and the risk of coronary artery disease (54). Polygenic risk scores (PRS) aggregate the effects of multiple genetic variants and show potential for predicting individuals ‘lifetime CVD risk, even in those with normal clinical parameters (54). Incorporating genetic screening and counseling into conventional risk assessment frameworks may improve early detection and inform personalized prevention strategies (55). Genomic profiling, when integrated with lifestyle interventions like diet optimization, smoking cessation, and physical activity, enhances precision cardiology to mitigate preventable CV events (55).

### Unhealthy foods

Global dietary patterns vary, with Asian populations typically adhering to high-carbohydrate diets, while Western populations tend to prefer high-fat, protein-rich foods (56). Excessive carbohydrate consumption is associated with dyslipidemia and vascular dysfunction, which increases the risk of CVD (57).

Research in health services increasingly endorses culturally tailored dietary interventions to mitigate region-specific nutritional risks associated with CVD (58). Substituting refined grains with whole grains, decreasing sodium consumption and encouraging diets rich in vegetables, fruits, legumes and unsaturated fats have shown significant advantages in lowering blood pressure, enhancing lipid profiles and decreasing CVD events. The findings from the PURE study revealed significant variability in dietary quality across different socioeconomic and geographic contexts, highlighting the need for interventions according to local food preferences and cultural traditions (59). Programs focused on community-based nutrition education and labeling have demonstrated effectiveness in encouraging healthier food choices, particularly in LMI (60). Sustainable dietary reform necessitates collaborative policymaking among dietitian experts, local governments, and health care systems to establish environments conducive to affordable, heart healthy food (61). Integrative approaches correspond with the global health agenda, focusing on non-communicable diseases prevention and equitable nutrition security.

### Social determinants of health

Social, economic, environmental, and psychosocial factors, collectively referred to as social determinants of health (SDoH), significantly influence cardiovascular outcomes. Low socioeconomic status (SES) correlates with chronic stress, unhealthy behaviors, restricted access to healthcare, and inadequate nutrition, all of which increase the risk of cardiovascular disease (CVD) (62). Neighborhood characteristics, including access to green spaces, safe walking areas, and recreational facilities, are associated with increased physical activity and reduced body mass index (BMI). Conversely, exposure to pollution and high-traffic environments is linked to a higher incidence of CVD.

Long-term cardiovascular benefits may result from addressing social aspects of health through transdisciplinary collaboration, equitable healthcare policies, and community-based interventions (63). Poverty, low educational attainment, unreliable housing, and insufficient access to care are aspects of structural unfairness that are known to drastically raise cardiovascular morbidity and mortality (64). The incorporation of Social Determinants of Health (SDoH) assessments into clinical practice, utilizing validated screening tools and social prescribing models, facilitates the linkage of patients to community resources. This approach has the potential to mitigate psychosocial stress and enhance treatment adherence (65). Health systems incorporating these practices into primary care frameworks report improved patient satisfaction, medication adherence, and blood pressure control (66). Additionally, initiatives that merge the social welfare, healthcare, and education sectors help reduce cardiovascular disparities in an approach that is sustainable (67). These approaches indicate an expanding field in preventive cardiology that combines public health equity with clinical practice.

### Aspects related to mental health and social interactions

Psychosocial stressors such as depression, job strain, social isolation, and perceived discrimination significantly contribute to the development of CVD (68). These stressors engage neuroendocrine and inflammatory pathways that expedite atherosclerosis and metabolic dysregulation.

**Figure 1:**
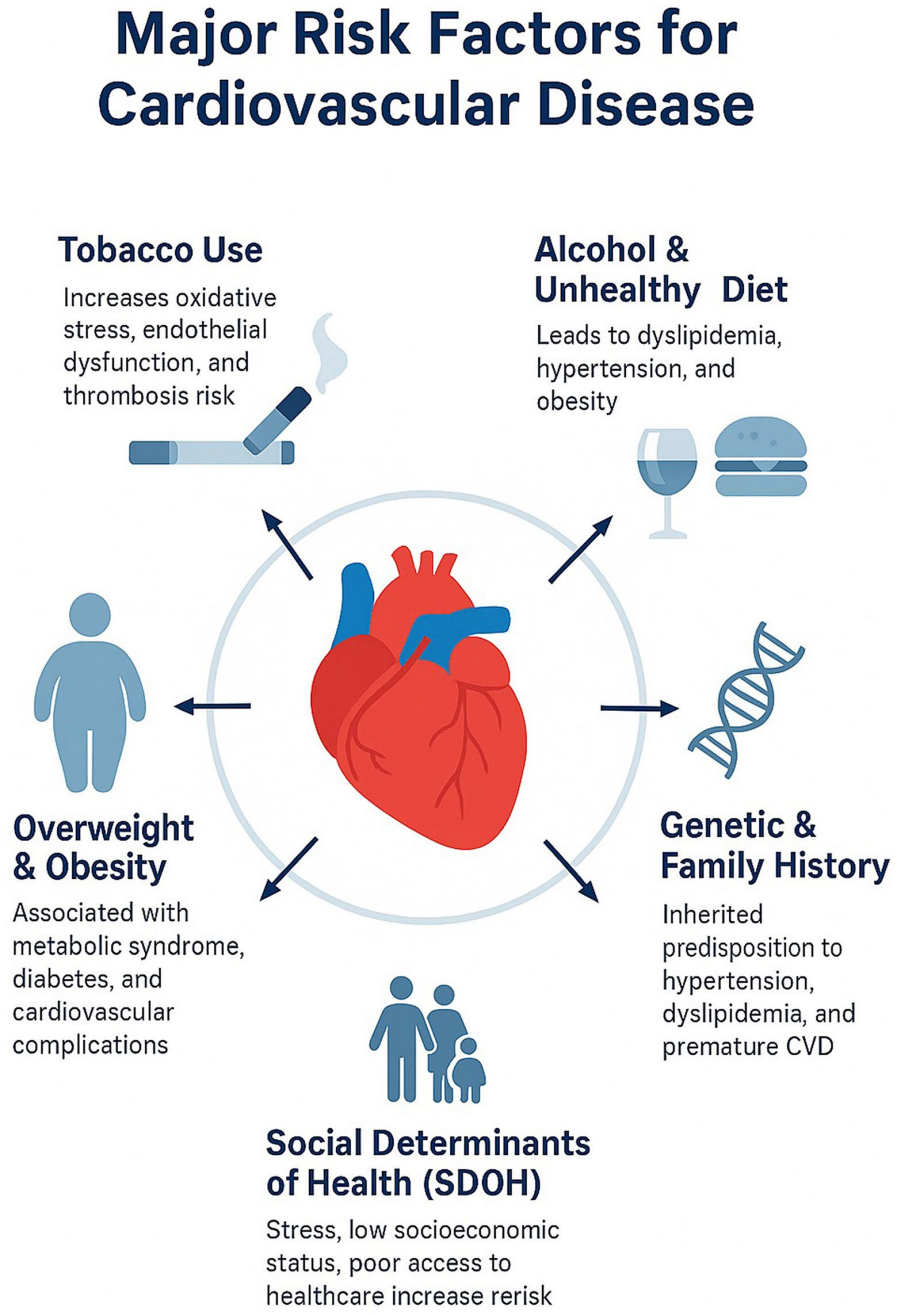
This figure highlights key modifiable and non-modifiable determinants contributing to CVD, placing the heart at the center to symbolize the primary organ affected by these factors. Determinants include smoking, which enhances oxidative stress, endothelial impairment, and thrombosis risk; unhealthy diet and drinking, promoting dyslipidemia, obesity, and hypertension; followed by obesity and overweight associated with metabolic syndrome and diabetes. Family history and genetic factors suggest a hereditary component to CVD. Lastly, SDoH, including low socioeconomic status, psychosocial stress, and inadequate healthcare accessibility, further exacerbate the risk of CVD. Collectively, these interconnected determinants underscore the multifactorial nature of CVD and reinforce the importance of coordinated and comprehensive integrated prevention strategies.

CVD is mainly caused by psychological triggers, particularly depression, work-related stress, social isolation, and perceived discrimination (69). Atherosclerosis and metabolic dysregulation are triggered by these triggers through the activation of neuroendocrine and inflammatory responses. Evidence-based interventions for psychosocial stress, including workplace wellness programs, community social support initiatives, and mindfulness-based stress reduction (MBSR), have demonstrated significant efficacy in reducing CV risk (70). Psychosocial determinants such as depression, chronic stress, and social isolation are considered distinct risk factors for myocardial infarction, hypertension, and stroke, acting through neuroendocrine and inflammatory mechanisms. Evidence from randomized trials indicates that MBSR positively influences CV health by enhancing overall mental health, blood pressure regulation, and heart rate variability among high-risk individuals (71). A very recent study suggested that programs promoting a healthy diet, physical activity, and a conducive workplace environment can help reduce cardiometabolic risk (72). Therefore, the integration of mental health care within CV prevention framework can improve overall well-being. Implementing such strategy further strengthens the CV prevention system. The various risk factors contributing to the development of CVD are illustrated in Figure 1.

### Strategies for the prevention of cardiovascular disease

A comprehensive strategy including clinical care, behavioral change and public health intervention is necessary to prevent CVD. Several studies reported that behavioral changes like quitting smoking, adequate sleep, following a healthy diet plan and regular exercise substantially reduce the CVD risk. Therefore, it is essential to implement CVD prevention programs that extend not only at individual level but also at community level by addressing social and structural inequities in healthcare facilities and social support systems (55). However, socioeconomic inequalities, healthcare accessibility, and regional variations persist globally as key factors in CVD outcomes (46). Therefore, comprehensive strategies that combine community involvement, population-level policies, and personalized risk reduction are increasingly recognized as a key to sustainable CVD prevention (73). Equitable health opportunities, an improved primary care system, and a strong social protection network at community level have shown promising results in lowering the CV morbidity in susceptible groups (74). In addition, rapid adoption of digital health tools, including mobile apps and telemonitoring systems, facilitates early diagnosis, therapeutic compliance, and supports virtual lifestyle management (75). Integrated care models that bridge primary healthcare with mental health play an essential role in promoting CV equity across populations.

### Quitting smoking for good

Tobacco use remains the primary cause of preventable deaths globally, despite numerous public health campaigns over the past several decades. Despite a decrease in global smoking rates from 42.6% in 1965 to 13.7% in 2018, relapse rates continue to be significant, with estimates ranging from 95 to 97% within 1 year of cessation attempts. Integrated cessation strategies that incorporate pharmacotherapy, such as nicotine replacement and varenicline, alongside behavioral counseling, have demonstrated the highest efficacy, enhancing abstinence rates by 10–20% (69).

Health systems must prioritize accessible and comprehensive smoking cessation services within primary care frameworks to mitigate the cardiovascular burden linked to tobacco use (39). Research indicates that integrating behavioral counseling, pharmacotherapy, and digital support tools significantly enhances quit rates relative to interventions that utilize a single component. Telehealth follow-ups facilitate ongoing patient engagement, particularly in rural and underserved areas, thereby promoting sustained tobacco abstinence (76). Community outreach initiatives, such as culturally tailored education campaigns and workplace-based programs, have shown considerable effectiveness in facilitating smoking cessation and preventing relapse. Integrating these services into universal health coverage frameworks improves accessibility and affordability, promoting equitable distribution across socioeconomic levels (41). This systems-level approach is consistent with global cardiovascular prevention strategies that prioritize health equity and population-level tobacco control (77).

### Regular exercise

Cardiorespiratory fitness (CRF), an indicator of cardiovascular and pulmonary health, is negatively correlated with all-cause mortality. Moderate to vigorous physical activity enhances cardiorespiratory fitness and reduces CV risk (78). Modest enhancements (1–2 METs) over a period of 3–6 months are associated with a 16–30% decrease in mortality (79). Meta-analyses have shown that the effectiveness of physical activity interventions in reducing CV events (80).

Urban design and policy reforms that facilitate active living via pedestrian-friendly infrastructure, secure bicycle lanes, and accessible green spaces are widely acknowledged as vital elements in the prevention of CVD (35). Research demonstrates that residents in walkable, transit-oriented areas have reduced incidences of obesity, hypertension, and coronary heart disease (81). Workplace wellness incentives and community fitness efforts promote ongoing participation in physical activity, enhancing cardiovascular fitness and mental health (82). Governments that allocate resources to public recreational areas and subsidized fitness initiatives have significant health and economic benefits, evidenced by decreased healthcare expenditures and improved productivity (83). These treatments align with the World Health Organization’s Global Activity Plan on Physical Activity (GAPPA), which aims for a 15% decrease in global physical inactivity by 2030 (84). Incorporating urban health initiatives into national development frameworks can produce significant long-term cardiovascular and societal advantages (85).

### Healthy foods

The scientific literature has demonstrated that Mediterranean and DASH dieting patterns are highly effective at reducing CVD activity and mortality because of large meta-analyses by (86, 87). Individuals who eat a heart-healthy diet, particularly the DASH and the Mediterranean diet, have a lower chance of CVD. Both diets give priority to whole grains, fruits, legumes, vegetables, unsaturated fats, and lean protein, limiting red meat, beverages that contain sugar and processed foods (88). Polyphage dietary interventions were also demonstrative of protective effects, and (89), reported a decline in CV mortality in several cohorts.

Interventions aimed at improving the dietary literacy of the population, accessible food labeling, and fiscal incentives are needed to improve adherence to cardio protective diets at the population level (90). Fruits, whole grains, and vegetables subsidies and taxation of sugar-sweetened beverages have demonstrated a substantial decrease in diet-related diseases such as CVD and type 2 diabetes (58). Dietary inequalities can be addressed through community-supported agriculture-based projects and local food systems, which can help to improve the availability of nutrient-rich foods to low-income groups (91). The fiscal and educational measures to change food environment are aligned with the WHO global strategy on diet, physical activity and health that emphasize the need for multisectoral collaboration to ensure sustainable nutrition equity (3). All of these measures will lower CV morbidity and build robust and healthy food systems (61).

### Optimal sleep

The duration and quality of sleep are recognized as modifiable risk factors for CV health. Sleep durations of less than 6 h and more than 9 h are associated with the risks of increased risks of high blood pressure, diabetes and CVD (92). The integration of sleep assessment into the framework of primary care and occupational health is also being recognized as a significant factor in CVD prevention (93). Poor or bad sleep is associated with metabolic disorders, hypertension, and systemic inflammation are more likely to develop MI and stroke (94). Therefore, regular monitoring of sleeping disorders like obstructive sleep apnea (OSA) facilitates early detection of individuals at risk and this can be very effective in determining CV outcomes (14). The integration of sleep timing, stress management training and cognitive-behavioral interventions into the medical and workplace wellness program offers an affordable mechanism for reducing cardiometabolic risk (92). Sleep monitoring technologies such as wearable sensors and home-based polysomnography, enable health systems to increase patient engagement and hence facilitate early detection of sleep abnormalities (95). These preventive strategies are in accordance with the health policy recommendations that emphasize the integration of lifestyle and behavioral risk management into a comprehensive CV care framework (77). A meta-analysis further supports the association between abnormal sleep duration and increased CV risk (94).

### Interventions targeting social determinants of health

Environmental and socioeconomic determinants have a significant impact on CV outcomes. Several studies have suggested that community-based interventions focusing on educational opportunities, housing quality, employment, and healthcare accessibility can help reduce CVD prevalence.

**Figure 2:**
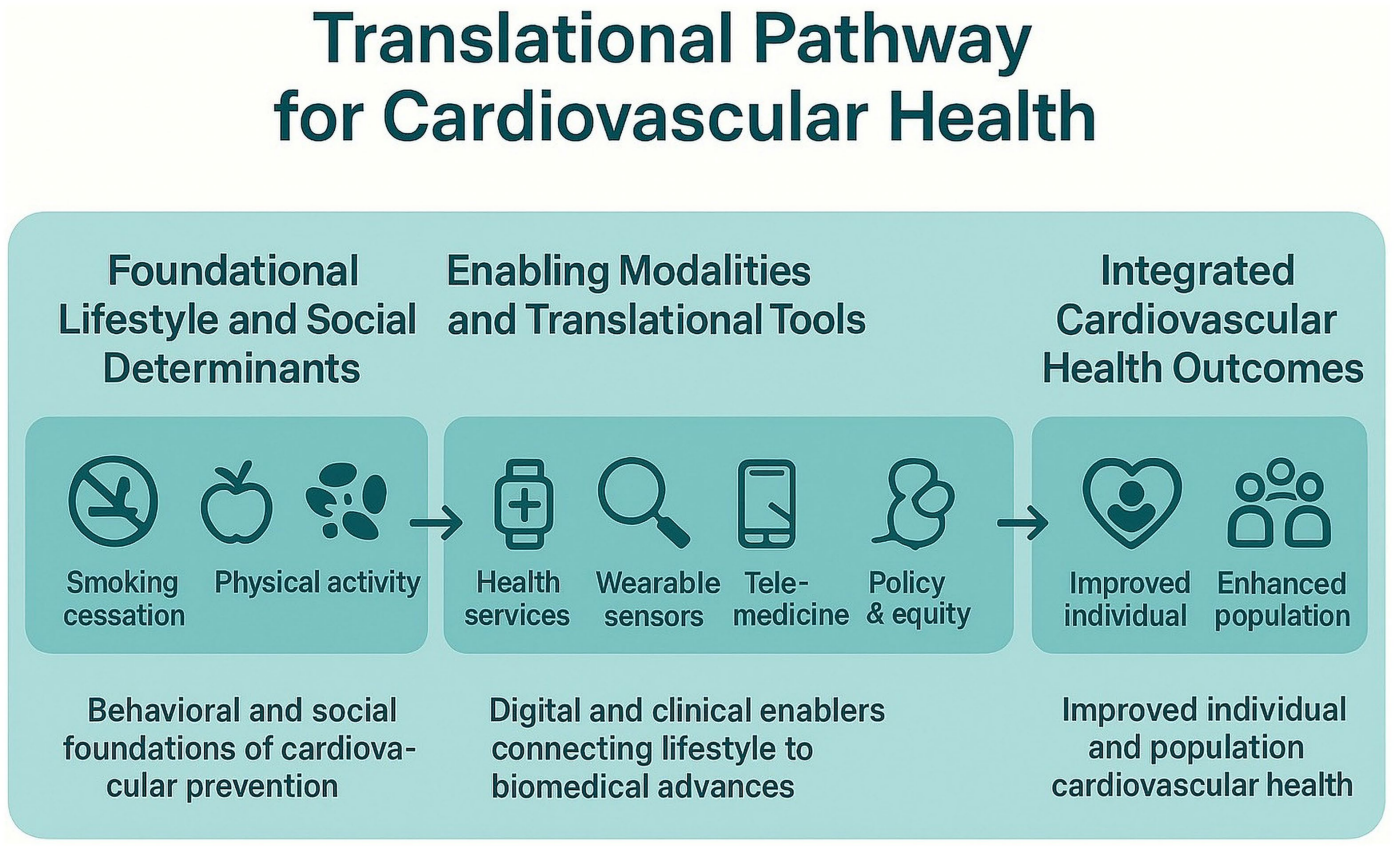
Shows the translational and progressive pathway of CV health from lifestyle behaviors and SDoH to advanced precision innovations and integrated outcomes. The pathway starts with lifestyle and SDoH determinants, emphasizing regular exercise, healthy eating, quitting smoking, and equitable access to healthcare. Followed by telemedicine, wearable sensors, and healthcare services that support continuous monitoring, early diagnosis and active patient engagement. In addition, CRISPR-Cas9 gene-editing technology is a promising therapeutic strategy for CVD prevention. The bidirectional arrows represent dynamic feedback among these frameworks, highlighting the translational flow and the significance of multidisciplinary collaboration.

The incorporation of SDoH into electronic medical records is increasingly recognized as a productive strategy to reduce the upstream factors contributing to CV health disparities (96). Integration of these systems helps clinicians in patient detection, having experience of socioeconomic barriers such as food insecurity, inadequate housing and unequitable healthcare access, thereby facilitating social and community support programs linkage (97). Recently, a study suggested that the integration of SDoH data within CV care framework enhances preventive interventions, chronic conditions and medication adherence. Furthermore, collaboration among clinicians, policy leaders, and community organizations are key in establishing such strategies that align with clinical care and social policy (97). Nations that have integrated these healthcare models into their national health frameworks have reported substantial advancements in reducing the CV disparities and overall population well-being (55). Therefore, it is necessary to develop intersectoral collaboration that ensures patient-centered care and social accountability (77). By adopting such preventive strategies, the risk of CVD can be minimized (Figure 2).

### Telemedicine/digital health innovations

Telemedicine has become a valuable tool for improving CV prevention and care, especially in the improvement of access to care and the provision of continuous monitoring. It has been demonstrated that interventions based on telemedicine can be successfully used to reduce blood pressure, improve lipid management, and increase adherence to treatment in a wide range of patients (19). Further possibilities of wearable sensors and mobile health apps enable real-time monitoring of biomarkers, such as physical activity, heart rate, and arrhythmias, among others, which are potentially significant in CV risk (17, 18). Multiple systematic reviews of telemonitoring have concluded that it leads to improved blood pressure regulation, as noted by (19). It has been demonstrated that these technologies are particularly useful in underserved and geographically isolated environments, where access can be facilitated and preventive health offered (98). According to a Cochrane review of (99), structured telephone support and telemonitoring decreases hospitalization and mortality in heart failure patients. Further systematic studies showed that mobile health interventions could have a considerable impact on the improvement of adherence and cardiovascular risk-factor management (100, 101).

The benefits of telemedicine, however, are not equally distributed. Internet connectivity, privacy, digital literacy barriers, and affordability of devices have an unequal impact on socioeconomically disadvantaged populations (23, 102). The other obstacles are the inconsistency between the accuracy of the devices and the varying regulations, which are making standard clinical adoption complex (103). The policy actions coordinated to address these gaps will involve equitable access to digital devices, standardizing the quality of devices, data security, and insurance to create a scenario whereby telemedicine helps in reducing (104), rather than exacerbate, CV health disparities.

## Genomic technologies: CRISPR in precision cardiology

### CRISPR-Cas9 in the prevention and treatment of cardiovascular diseases

Genetically mediated CVD, including congenital heart defects, familial hypercholesterolemia, and inherited cardiomyopathies, has emerged as a potential target for gene editing technologies like the CRISPR-Cas9 system. This technology enables the precise modification of disease-related genes, transforming experimental CV research by making gene editing more efficient and cost-effective.

At the population level, the CRISPR-Cas9 system has implications both for public health and clinical practice. Theoretically, a combination of genetic risk assessment and gene-based interventions can allow early identification of those individuals who carry high-risk variants and thereby facilitate timely interventions by lifestyle modification and pharmacotherapy (105, 106). However, the translation of this technology into clinical practice is limited and requires strict ethical regulations, robust safety evaluation, transparent data governance, and sustained public trust. Health economists also emphasize the need for cost-effectiveness analysis, especially in LMI, to prevent global inequalities in access to this genomic advancement (107). Policies that interconnect genomic research, health-care facilities, and global health will be essential in the responsible implementation of CRISPR technology.

### Targeting inherited cardiomyopathies

Inherited cardiomyopathies, such as hypertrophic cardiomyopathy (HCM), arrhythmogenic right ventricular cardiomyopathy (ARVC), and dilated cardiomyopathy (DCM), are mostly caused by mutations in sarcomeric genes. In an experimental model, CRISPR-Cas9 technology has been used to inactivate pathogenic alleles, aiming to restore normal gene function. For example, editing of PRKAG2 gene in preclinical models of Wolff-Parkinson-White syndrome has improved the cardiac structure and rhythm (108).

These findings suggest that CRISPR-based therapies can be a part of precision cardiology in the near future, especially when combined with early diagnosis, family screening and longitudinal follow up via electronic health records (109, 110). This approach perfectly aligns with precision public health concepts at a broader level, where integration of genetic information with clinical and environmental data to suggest more targeted prevention and treatment strategies (111). However, this technology is still in the experimental phase and is not yet part of standard CV care.

### Therapeutic strategies for the loss of function of mutations

Loss-of-function mutations in autosomal recessive genes or X-linked genes are implicated in a variety of CVD such as cardiomyopathy related Duchenne muscular dystrophy (DMD). CRISPR-based exon skipping or deletion strategies have effectively restored the levels of dystrophin in cellular and cardiac models and were able to enhance the performance of human induced pluripotent stem cell (iPSC)-derived cardiomyocytes (112). These findings are consistent with the possibility of CRISPR-Cas9 in treating otherwise incurable inherited cardiomyopathies. The implementation of such therapies in clinical practice needs to be closely dedicated to involving regulators, payers, clinicians, and ethics boards to ensure their safety, equity, and sustainability (113). Regulatory principles that are suggested by the agencies, including the US Food and Drug Administration (FDA) and the European Medicines Agency (EMA), focus on off-target effects, long-term safety, and effective procedures of informed consent (114). Health technology assessment, such as cost-effectiveness analysis, will become important to make sure that genomic interventions can be prioritized by the needs of public health, and not only niche, high-cost interventions (115). Ethical regulation, openness, and consultation will be at the center stage as gene-editing methods become nearer to clinical application (116). Therefore, health service researchers, policy-makers and bioethicists should collaborate to produce a balanced model that would promote innovation and at the same time guarantee patient welfare and equity in society.

### Precision gene correction and ethical considerations

CRISPR-Cas9 itself, in principle, can cause disease-relevant mutations on a drop-in basis with high precision; however, in reality, the practicability of this correction effort varies dramatically across cell types and disease conditions. Some studies have demonstrated successful gene repair in cardiac tissue and even in embryos at early stages (117, 118); however, germline editing raises significant ethical, social, and legal concerns, as genetic alterations can become inherited by future generations.

As a result, recent studies and initial clinical trials principally involve somatic gene editing, which marks non-reproductive cells and does not involve mechanisms of heredity (119, 120). Safety, off-target and immune responses in long-term studies are subject to registries and movements across national borders and countries, and such studies will be a requirement in the first in human applications (105). Researchers and policymakers should devise explicit processes to address informed consent, data privacy, justifiable access, and universal representation in gene-editing studies (121). To make sure that genomic innovation will develop in the context of equity and social responsibility, a transdisciplinary approach, i.e., the connection between clinical science, proposing the idea of the common good, and, thus, ethics and law, will be necessary.

### Preventing CVD before onset

Technological setting: One of the most promising uses of CRISPR-Cas9 in prevention is targeting genes with strong effects on the lifetime risk of CVD. PCSK9 gene editing in preclinical models, including but not limited to, has resulted in long-term reductions in low-density lipoprotein cholesterol (LDL-C) levels of about one-third, with a corresponding reduction in atherosclerotic lesion formation (122, 123). This idea is because it can drastically change the paradigm of CVD prevention, whereby one gene-based intervention could replace decades of lipid-lowering medication.

But, until such methods could be commercialized into clinical practice, critical concerns, like the safety levels in the long-term, off-target effects, ethical control, cost-effectiveness, and health-system capacity, had to be effectively addressed (119, 121). Policy development that relies on bioethics and assessment of health technology will become instrumental in integrating these therapies in a manner that does not exacerbate the current health disparities.

### Preventing congenital and inherited heart conditions

CRISPR-Cas9 can also contribute to the prevention of some CV disorders, such as congenital heart disease (CHD). CRISPR-based editing has avoided glycogen accumulation and myocardial tissue structural abnormalities in experimental PRKAG2-associated models in cardiac syndrome (24). In the future, applications may include neonatal genomic screening in combination with somatic gene therapy or neonatal genetic interventions, as well as access to assisted reproductive technologies (ART) (124). The responsible implementation of these technologies will be based on inclusive governance frameworks that prioritize clinical safety, ethical integrity, and equitable access to various health systems through the deployment of the potentially transformative CRISPR-Cas9 and other methods, combining conventional and emerging strategies to manage CVD. Table 1 summarizes the major lifestyle risks, social and economic determinants, telemedicine, and genomic interventions, illustrating how conventional and emerging strategies intersect in the prevention and management of CVD.

**Table 1:** Key studies on modifiable risk factors, social determinants, telemedicine, and emerging genetic therapies in cardiovascular disease.

Category	Study/topic	Key findings	References
Modifiable risk factors	Smoking (including electronic cigarettes & passive exposure)	Both active and passive smoking increase CVD risk; electronic cigarette use is linked to cardiovascular dysfunction and platelet abnormalities.	(8, 38, 129, 130)
	Obesity & lifestyle (BMI, diet, and alcohol consumption)	Overweight/obesity (BMI ≥ 25) increases risk of hypertension, T2DM, and CVD; healthy BMI (20–24.9), balanced diets (Mediterranean/DASH), and moderate alcohol intake reduce risk; heavy drinking increases atrial fibrillation, cardiomyopathy, and stroke.	(9, 42, 43, 131)
	Sleep (duration & disorders)	Both short and long sleep durations, and disorders such as OSA, increase the risk of hypertension, diabetes, coronary disease, and arrhythmias; the optimal duration is 7–8 h.	(92, 94, 132)
Social determinants	Socioeconomic status (SES, healthcare access, built environment)	Low SES contributes to chronic stress, limited healthcare access, poor nutrition, and reduced activity, all of which increase CVD risk and worsen outcomes.	(31, 62, 74, 83, 97)
Telemedicine	Digital health wearable sensors etc	Telemedicine is a valuable tool for enhancing CV prevention, Digital literacy barrier, limited internet facility, and data privacy	(17–19, 75, 101, 102, 104)
Emerging genetic therapies	CRISPR-Cas9 for inherited cardiomyopathies	Allele-specific editing of disease-causing mutations (e.g., PRKAG2, RYR2) improves function and corrects arrhythmias in preclinical studies.	(123)
	CRISPR for loss-of-function mutations (e.g., Duchenne muscular dystrophy)	Exon deletions/restorations restore dystrophin expression and improve cardiac/muscle function in preclinical models and iPSC-derived cardiomyocytes.	(112, 122)
	PCSK9 base editing for atherosclerosis prevention	CRISPR-mediated PCSK9 editing reduces LDL-C durably (35–40% in mice; sustained effects in primates), advancing toward early clinical trials.	(24, 123)

### Ethical, regulatory, and policy perspectives

The implementation of the CRISPR-Cas9 technology in CV medicine must have a reasonable balance between its scientific role and the ethical obligation. The development of multidisciplinary oversight committees by health systems is recommended when gene-editing therapies are transferred to clinical practice. Such committees, which should comprise clinicians, molecular geneticists, bioethicists, policymakers, and patient representatives, have ensured that translation research and therapeutic uses align with the key principles of bioethics: autonomy, beneficence, non-maleficence, and justice (121). These committees must ensure that protocols are clear, patients make a meaningful informed consent, risks and benefits are properly assessed, and long-term follow-ups after the trial are established (120). In this way, the interplay of ethical factors must be established in national health-technology appraisal mechanisms and institutional review boards (IRBs) to foster confidence in the population and promote fair access to innovative technologies, thereby avoiding their mistreatment or unjustified use (125). Accordingly, CRISPR-based therapeutic interventions in cardiovascular diseases require governance arrangements that prioritize scientific standards, patient safety, as well as moral responsibility.

Notably, ethical issues are not limited to genome editing, but also apply to other emerging technologies reviewed in this paper, including telemedicine, mobile health applications, wearable sensors, as well as lifestyle-modification programs. These technologies raise concerns about digital surveillance, information privacy, algorithmic bias (126), monetization of health data, and disparities among digital infrastructures. It is crucial in the public health interest to ensure informed consent in remote monitoring and protect sensitive biometric data, as well as to eliminate the exclusion of individuals with low digital literacy and limited internet access facilities (23).

Notably, hegemonic regulatory organizations have already suggested tangible governance systems to address responsible execution. The WHO has published a Global Governance Framework for Human Genome Editing, emphasizing transparency, openness, fairness, and the ban on unlawful or dangerous applications (127). Furthermore, the US National Institutes of Health (NIH) governs the study of gene-editing by using its NIH Guidelines to Research Involving Recombinant or Synthetic Nucleic Acid Molecules, which are supervised by the Novel and Exceptional Technology and Research Advisory Committee (NExTRAC), which requires strict ethical supervision, trial review, and publication of research (128).

## Conclusion

Interacting behavioral, social, and genetic factors contribute to the development of CVD. This narrative review reveals that lifestyle modification is the most effective approach in the context of risk reduction for CVD, and digital health technologies, including telemedicine and wearable devices, have the potential to enhance monitoring, adherence to treatment, and care provider. Newer genomic technologies have presented opportunities, with some, such as CRISPR-Cas9, promising avenues for treating inherited cardiovascular diseases; however, application remains restricted by safety, ethical, and equity implications.

The new gaps in especially important research are the long-term efficacy of digital-health interventions in heterogeneous populations, how to maintain behavioral change, and an all-encompassing consideration of CRISPR-based therapies. Ethically controlled, fair, and interdisciplinary measures to address these gaps will be crucial in ensuring a significant reduction in CVD load worldwide.

## Limitations

There are a number of limitations to this narrative review. Since it is not a systematic review, relevant studies might have been overlooked in the search strategy and the synthesis is liable to interpretation by the author. There is a risk of publication bias, which is especially likely in the case of digital-health and CRISPR-related research, where only positive results are often reported. There is also a wide range of evidence reviewed by different populations, study designs and types of interventions, which introduces significant heterogeneity and cannot be compared directly. These are some key aspects that should be considered when interpreting the conclusions.

## Implications for policymakers and clinicians

This narrative review has several practical implications for the findings presented herein. Lifestyle counseling, digital monitoring systems, and early genetic risk must be incorporated in routine care as a time-saving tool to improve prevention in high-risk populations. Policymakers are encouraged to invest in a fair digital health infrastructure, boost data privacy, and preventive programs at the community level. There is also a need for clear regulatory frameworks to safeguard the safe and ethical use of emerging technologies such as CRISPR-Cas9. A solution involving coordinated efforts among healthcare organizations, governments, and community organizations will be necessary to transform behavioral, digital, and genomic innovations into scalable and equitable CV health actions.

## Future perspective

1. The future of CV care depends on integrating public health prevention with genomic insights, enabling the adoption of such interventions so that communities and individuals to benefit from truly personalized and precision-based strategies.
2. Emerging CRISPR-based therapies offer transformative potential to correct inherited mutations at their source, redefining CVD care from managing disease to preventing it before it begins
3. Advancing these innovations in clinical practice needs strong ethical oversight, unified regulatory standards, equitable access, and interdisciplinary collaboration to promote CV health globally.
